# Ozone/Thiosulfate-Assisted Leaching of Cu and Au from Old Flotation Tailings

**DOI:** 10.3390/molecules30010069

**Published:** 2024-12-27

**Authors:** Stefan Trujić, Miroslav P. Popović, Vesna Conić, Miloš Janošević, Filip Alimpić, Dragoljub Bajić, Ana Milenković-Anđelković, Filip Abramović

**Affiliations:** 1 Department of Environment and Sustainable Development, Singidunum University, Danijelova 32, 11010 Belgrade, Serbia; stefan.trujic@irmbor.co.rs (S.T.); amilenkovic.andjelkovic@singidunum.ac.rs (A.M.-A.); filip.abramovic@eko.gov.rs (F.A.); 2 Mining and Metallurgy Institute Bor, Albert Ajnštajna 1, 19210 Bor, Serbia; vesna.conic@irmbor.co.rs (V.C.); milos.janosevic@irmbor.co.rs (M.J.); 3 Faculty of Mining and Geology, University of Belgrade, Đušina 7, 11120 Belgrade, Serbia; dragoljub.bajic@rgf.bg.ac.rs; 4 Ministry of Environmental Protection, Government of Serbia, Omladinskih Brigada 1, 11070 Belgrade, Serbia

**Keywords:** leaching, composite, thiosulfate, ozone, tailings, XRD, SEM, polarizing microscopy

## Abstract

The growing demand for metal production promotes the search for alternative sources and novel modalities in metallurgy. Flotation tailings are an important secondary mineral resource; however, they might pose a potential environmental threat due to containing toxic metals. Therefore, proper leaching reagent selection is required. Ozone is an alternative oxidizing agent for metal leaching, as its use prevents contaminating product generation while increasing the noble metal extraction efficiency in the presence of complexing agents. In this study, the feasibility and efficiency of combining the use of thiosulfate and ozone for gold and silver extraction have been investigated as an eco-friendly alternative for recovery from flotation tailings. Two sets of samples from old flotation tailings of Copper Mine Bor (Serbia) were prepared and physico-chemically characterized, then treated in two experimental leaching procedures, followed by thorough XRD and SEM/EDS analyses of the products. It showed that after 1 h of leaching in a water medium at room temperature and a solid-to-liquid phase ratio of 1:4, 88.8% of Cu was obtained, while a high efficiency of Au extraction from solid residue (after Cu leaching) was attained (83.4%). The results suggest that ozone-assisted leaching mediated by Ca-thiosulfate can be an effective eco-friendly treatment for noble metals recovery from sulfide-oxide ores.

## 1. Introduction

The growing demands for intense metal production on one hand, and the rapid spending and degradation of primary mineral deposits on the other, impose the search for alternative solutions of metallurgical processing. This is particularly important for metals with a broad range of uses, such as copper [1]. Mining activities have a detrimental effect on the environment, reflected in land area degradation, the disposal of enormous solid waste, and land acidification, which is common for copper extraction from sulfide ores [2,3,4]. A majority of the world’s copper production comes from copper–iron–sulfur minerals. One of the important technologies for secondary raw material treatment is froth flotation, in which the hydrophobic particles are attached to air bubbles and floated back to concentrate, while the hydrophilic particles remain in the liquid phase [5]. In it, sulfide minerals of the valuable metals are being concentrated, leaving gangue minerals in the tailings. The mineral concentrate, which contains, on average, 20–30% of copper, is further processed in smelters in order to produce pure copper. Sulfide ore processing is followed by a noticeable loss in non-ferrous and precious metals (ending into wastes) but is also a technogenic source of metals [6,7]. Flotation tailings, as a type of mining waste generated in the mineral concentrate production from the ore, pose another environmental problem, being ~ 99% (by weight) of the processed ore and potentially containing various toxic or heavy metals [8,9]. On the other hand, they might be a valuable secondary mineral resource. The deposition of metals in flotation tailings can be significant as a consequence of the vast amount of waste accumulated over decades of mineral resource exploitation [7,10,11]. The flotation tailings of sulfide ore, such as those containing pyrite with impurities of various non-ferrous metals, are notable causes of acid mine drainage. This feature is generated mainly in the process of sulfide oxidation induced by water and oxygen availability [7]. Sulfide oxidation leads to the formation of secondary minerals, such as sulfates (gypsum, poitevinite) and hydroxylated sulfates (jarosite, alunite), and metal cations and sulfate anions release into the liquid phase [12,13].

There have been different hydrometallurgical approaches and agents investigated and developed in recent decades that can mediate the extraction of metals from mineral raw materials, such as acid leaching (hydrochloric, sulfuric, organic) [3,14], salt leaching (by Na- or Cu-chloride, etc.), base leaching (ammonium salts, cyanides) [15], various bioleaching methods [9], flotation [12], and oxidation methods. Bioleaching is a highly promising method, but its drawbacks are often slow kinetics and protracted leaching time [16]. Ozone as a strong oxidizing agent can be used for metal extraction, as its use does not contaminate the processed products and does not generate hazardous waste. This is particularly explored as a safer approach for gold and silver leaching from ores and as a powerful alternative to the pretreatment of refractory gold ores [17], pivoting toward environmental stewardship [Torres 2016]. The oxidation capacity of ozone is high enough (2.7 V) that it is able to oxidize all metals and sulfides and can be used at concentrations as low as 10^−4^ M [17]. There are a number of studies on the use of ozone for dissolving noble metals in mineral acids [18,19] and for the extraction of various transition metals [20,21,22]. It has been shown that ozone oxidation is more effective than bio-oxidation in the case of sulfide ore (pyrite) [23], while there is a triple increase in Au and Ag recovery (from 9% to almost 30%) after ozone pretreatment [24]. Advanced procedural modalities have been developed, including the ozone-assisted cyanide and thiocarbamide leaching of noble metals from mineral raw materials [24,25]. The improvement in overall performance efficiency has been confirmed, referring to both (1) the increase in extraction efficiency (of metals into solution) and (2) the increase in selectivity of extraction from complex mixtures, while the decrease in process duration has been addressed [19]. The efficiency of metal leaching is directly proportional to the applied ozone dose [17].

In the absence of complexing agents in solution, Au starts dissolving at potentials beyond 1.3 V [26]. Although moderate at lower potentials, complexing agents stimulate oxidative dissolution, moving the dissolution onset potential to less positive values, thus enabling leaching [27]. Similar insights were obtained for Ag with ozone and thiosulfate [28,29]. The common complexing agents in Au and Ag leaching are cyanide and thiosulfate anions in alkaline media and Cl^−^ in acidic media [30,31,32]. Thiosulfate is an alternative lixiviant to cyanide, being highly selective toward gold under mild conditions [33,34] and, at the same time, is a promising alternative to the highly toxic cyanidation process. However, thiosulfate-based gold leaching is not yet in large-scale application due to its drawbacks: the gold recovery is lower compared to that in other processes [33], the thiosulfate consumption is high [34], and it favors the formation of a resistant passivation layer [27].

The following reactions have been proposed for gold oxidation by ozone [27]:

(1) $$Au+\frac{1}{2}O_{3}+H^{+}\to {Au}^{+}+\frac{1}{2}O_{2}+\frac{1}{2}H_{2}O$$

(2) $$Au+\frac{3}{2}O_{3}+{3H}^{+}\to {Au}^{3+}+\frac{3}{2}O_{2}+\frac{3}{2}H_{2}O$$

On the other hand, the aurous Au^+^ and Au^3+^ ions form a complex with S_2_O_3_^2−^, which keeps them in ionic form and preserves them from reduction, or precipitation (in the form of oxides/hydroxides) [27,35]:

(3) $${Au}^{+}+{2S}_{2}O_{3}^{2-}\to {\left[Au{\left(S_{2}O_{3}\right)}_{2}\right]}^{3-}$$

(4) $${Au}^{3+}+{2S}_{2}O_{3}^{2-}\to {\left[Au{\left(S_{2}O_{3}\right)}_{2}\right]}^{-}$$

The equations for silver would be analogous to Equations (1) and (3) (i.e., for Au/Au^+^), though at a different potential:

(5) $$Ag+\frac{1}{2}O_{3}+H^{+}\to {Ag}^{+}+\frac{1}{2}O_{2}+\frac{1}{2}H_{2}O$$

(6) $${Ag}^{+}+{2S}_{2}O_{3}^{2-}\to {\left[Ag{\left(S_{2}O_{3}\right)}_{2}\right]}^{3-}$$

The dissolved gold experiences passivation partly due to the presence of various compounds (ammonia, humic acids, etc.) and cations in the leachate (Ag, Fe) but mainly due to the accumulation of sulfur coatings from thiosulfate decomposition on the gold surface [35,36]. In order to prevent this degradation, it is noticed that the use of CaS_2_O_3_ (unlike sodium- or ammonium-thiosulfate) supports the gold extraction, as Ca prevents the thiosulfate degradation on the gold surface, thus maintaining a high leaching rate during the process [37].

A literature overview gives evidence that the vast majority of studies focusing on oxidation-assisted Au/Ag leaching deal with non-thiosulfate lixiviants such as cyanide and ammonia, while those dealing with thiosulfate are rare [35,38,39,40]. There is, however, an evident lack of the use of ozone combined with thiosulfate agents for Au leaching. Hence, the objective of this study is to investigate and evaluate the feasibility and efficiency of combining the use of thiosulfate and ozone for gold and silver extraction as a novel alternative for environmentally conscious practices in the recovery of valuable metals from flotation tailings.

The subject of this study is the old tailing pond at Copper Mine Bor (Serbia), which has been deposited on one open pit site for over 70 years and contains nearly 24 million tons of reserves. It represents a significant environmental pollutant for the area surrounding the Bor mines due to containing acid pollution reagents with pH ~ 2 and fine particles of the tailing as dust. However, it also contains 2–3% of unused Cu remains, around 0.38 g/t of Au, and 2.27 g/t of Ag. In the dry seasons, this tailing causes dust in the surroundings, while in the rainy seasons, it causes mud on site and pollutes underground waters. As it is fine-grained, it is suitable for further hydrometallurgical treatment.

## 2. Materials and Methods

### 2.1. Methods of Sampling and Sample Preparation

The old flotation tailings from the dump located in the Copper Mine Bor (Bor, Serbia) were used for leaching and subsequent analyses. The dump contains ~2.8 × 10^7^ t of flotation tailings separated in Field I and Field II. Field I contains more copper (~0.2 wt%), mainly in form of sulfates and oxides (with oxide-to-sulfide ratio ~ 1.5:1.0). Field II contains mostly sulfide minerals; its copper content is ~0.1 wt%, and its oxide-to-sulfide ratio is ~1:4 [9]. The content of Au and Ag in flotation tailing is 0.3–0.35 g/t Au and 2.17–2.5 g/t Ag, respectively [41]. Copper concentration at the surface of the dump is only ~0.026 wt% and increases with depth; at 20 m, it is ~0.43 wt% [9].

A total of 455 samples were collected from depths from 0 to 63.6 m from 16 locations from Field I and Field II of the old flotation tailings dump and put in plastic bags, with each having a mass from 8.5 to 17.5 kg. The samples from Field I and Field II were labeled “Composite I” and “Composite II”, respectively. Subsamples were taken from each bag in the laboratory (by coning and quartering method) and were mixed in order to make one composite sample.

### 2.2. Initial Physical and Chemical Characterization of the Composite Samples

The density of particles and the bulk density have been determined according to the European standards EN 1097-6 [42] and EN 1097-3 [43], respectively. The acidity (pH) of the solution was monitored using a combined pH electrode Aqua Lytic SD300, described elsewhere [44]. Granulometric analysis (particle size distribution) of the two composite samples of flotation tailings has been performed by querying method (using laboratory sieves made of thin net, wire, and perforated metallic plate), according to the national standard SRPS ISO 2591-1:992 [45].

For chemical analyses of the Composite I and II samples, 10 g of each composite flotation tailings sample was dissolved in aqua regia. After the dissolution of a solid sample, the concentration of Cu, Fe, Ag, and Au was determined by Perkin Elmer Analyst 300 (Perkin Elmer, Norwalk, CT, USA) atomic absorption spectrophotometer (AAS), while Ca, K, Na, Zn, As, Sr and Al_2_O_3_ were determined by SPECTRO-Germany (Kleve, Germany) inductively coupled plasma atomic emission spectroscopy (ICP-AES). Sulfur has been analyzed by Leco and sulfur analyzer (ACS), and thermogravimetric analysis (TGA) was used to determine SiO_2_ content. TGA was performed using an SDT Q600 V20.9 Build 20 instrument (TA Instruments, Milford, MA, USA) operating in the temperature range of 25–700 °C and in a stream of nitrogen (at 100 mL/min flow rate and 10 °C/min heating rate) using a ceramic pot.

### 2.3. Instrumental Analyses (Mineralogical Characterization)

#### 2.3.1. Polarizing Microscopy

A polarizing Carl-Zeiss “Axioscope 5” microscope (Carl-Zeiss, Oberkochen, Germany) for reflected and transmitted light, equipped with “Image Acquisition Zeiss –Axiocam 105 Colour” system for photomicrography (Carl Zeiss, Germany), was used for mineralogical analysis.

#### 2.3.2. XRD Analysis

X-ray diffraction (XRD) analysis was performed by “Rigaku MiniFlex 600” instrument produced in Japan, equipped with a “D/teX Ultra 250” high-speed detector and an X-ray tube with a copper anode (Rigaku, Tokyo, Japan), with detection limit of ~1%. The recording conditions were as follows: 3–90° angle range, 0.02° step, 10°/min recording rate, and 40 kV /15 mA tube’s voltage and current (respectively). Mineral identification was performed using PDXL 2 Version 2.4.2.0 software, and the obtained diffractograms were compared to the data from the COD database.

#### 2.3.3. SEM/EDS

In order to perform an electron microanalysis, a small amount has been sorted out of each powdered composite sample (of 75 to 40 μm mean particle size), then mounted in “Allied EpoxySet” epoxy holder mixture (resin: hardener = 25:3 wt. ratio). After being frozen in epoxy (~10 cm^2^ surface area), these samples were polished by SiC paper of 30, 20, and 15 μm, then by 6.0, 3.0, and 1.0 μm diamond suspensions, and eventually cleaned with ethanol and acetone.

Samples were carbon-coated in AGAR Automatic Carbon Counter for SEM AGB7367A chamber (Agar Scientific, Essex, UK) and then analyzed using JEOL-IT 300 scanning electron microscope (SEM) (JEOL, Tokyo, Japan) equipped with Aztec (version 3.1.) energy-dispersive X-ray spectrometer (EDS). Elemental analyses were performed at 20 kV acceleration, by using a standard, and contents normalized to 100%. The detection limit of analyzed elements was ~0.1 wt%. Each obtained spectrum was described and discussed, and results are presented in wt%. The samples analyzed by SEM/EDS were solid residues from Cu and Fe leaching.

### 2.4. Experimental Procedures

#### 2.4.1. Cu Leaching—Experimental Protocol 1

**Figure 1:**
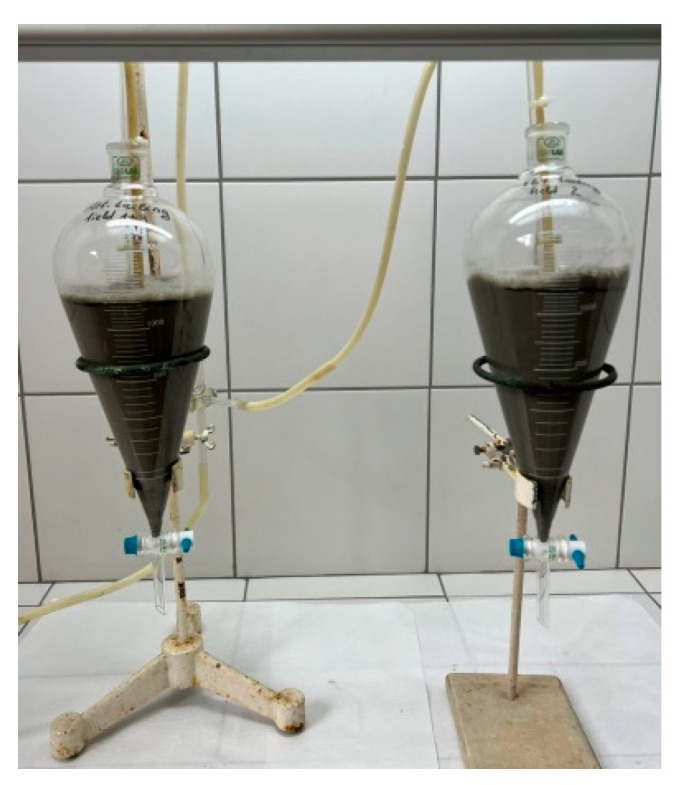
Separatory funnels for ozone leaching of Composite I and Composite II samples.

Leaching of flotation tailings of the Composite I and Composite II samples has been performed by water accompanied by ozone adding, respectively in separatory funnels shown in Figure 1. A 15% suspension (of solid-to-liquid phase ratio being 1:6.66) has been prepared. Ozone flow rate was put at 3 L/min per 2 samples, at 80% capacity. Mass of the leaching sample was 187.5 g (per experiment), while water volume for leaching was 1225 mL (per experiment). The concentration of dissolved Cu and Fe was measured along with the redox potential and pH value in the experiment. Total leaching time was 56 h, with sampling performed every 8 h. After Cu leaching, solid remnants were dried, homogenized, and weighed.

#### 2.4.2. Cu Leaching—Experimental Protocol 2

As there was a relatively high copper leaching from Composite I sample obtained after 8 h of experiment duration by protocol 1, another experiment was conducted to analyze leaching in a span of 8 h. The solid-to-liquid phase ratio in this experimental protocol was 1:4 in order to increase the Cu concentration in solution. Ozone flow rate was 3 L/min per 2 samples, at 80% capacity. Mass of the leaching sample was 100 g (per experiment), while the water volume for leaching was 400 mL. Sampling was performed after 1, 2, 4, 6, and 8 h (during the total of 8 h of experiment), and concentration of dissolved Cu and Fe was measured.

The optimization in ozone delivery for the leaching process was attained by utilizing about 13 g/m^3^ of O_3_ in the experiment, whereas the residuals were O_2_ and N_2_ from the air, leading to the energy consumption of approximately 100 Wh. The flow rate of existing gas from apparatus was about 5 L/min.

#### 2.4.3. Au and Ag Leaching from Solid Residue

After copper leaching in experiment 1, solid residues of Composite I and Composite II samples were used for Au and Ag leaching in thiosulfate solution. The parameters and conditions were selected based on the authors’ experiences from previous research on similar resources, i.e., based on the best results demonstrated in gold leaching processes. The selected parameters/conditions were ozone as an activation agent (flow rate of 3 L/min, at 80% capacity), Na_2_S_2_O_3_^2−^ (50 g/L) as a lixiviant for gold and silver, CuSO_4_·5H_2_O as a catalyzer (1:5 solid-to-liquid ratio), and leaching time of 48 h at room temperature. After process completion, the obtained pulp was filtrated, and solid residues were chemically analyzed to determine the range of leaching (i.e., leaching efficiency).

By analyzing the previous investigations on leaching various Cu concentrations with ozone-enriched air (conducted at Mining and Metallurgy Institute, Bor), the optimal ranges of parameters in ozone leaching process were determined and used subsequently in this pilot study. The next study plans to examine the effects of varying ozone flow rate, pH, and thiosulfate concentration on leaching rate and efficiency of Cu, Fe, Au, and Ag.

## 3. Results

### 3.1. Results of Physical and Chemical Characterization of the Composite Samples

The basic properties (apparent density, bulk density, acidity) have been found to be very similar in the two composites (I and II), as shown in Table 1. Based on the granulometric analysis results, it has been found that 66.5% of the particles in the Composite I sample and 69.1% of the particles in the Composite II sample have a diameter <75 µm (see Table 2 and Table 3, respectively).

The chemical composition of the representative samples from the two flotation tailings’ composites is given in Table 4. Based on the results, the percentage of Cu-oxides in the total copper ((Cu)_ox_/(Cu)_tot_) in the analyzed composites is 0.18/0.25 = 72% (Composite I) and 0.12/0.23 = 52% (Composite II).

### 3.2. Polarizing Microscopy of the Representative as-Received Samples from the Old Flotation Tailings

Based on polarizing microscopy analysis, a high content of pyrite (97–98% of the total sulfide mass) and a very low content of copper sulfide minerals have been detected in the flotation tailing samples. Digenite, covelline, and enargite are the dominant minerals in Composite II (~2% of total sulfide mass), while enargite and chalcopyrite are most abundant in Composite I (~3% of total sulfide mass). Free minerals of gold (electrum) are detected in the Composite I sample. The most abundant minerals in tailings are silicates and quartz, while carbonates and sulfates are less abundant. Copper oxides are part of the Cu-limonite mineral. The sulfide minerals of copper in samples (detected in traces) were covelline, digenite, and enargite. The mineral content in the analyzed samples is given in Table 5. Pyrite is the most abundant sulfide mineral detected in the samples.

### 3.3. XRD Analysis of the Representative as-Received Samples from the Old Flotation Tailings

**Figure 2:**
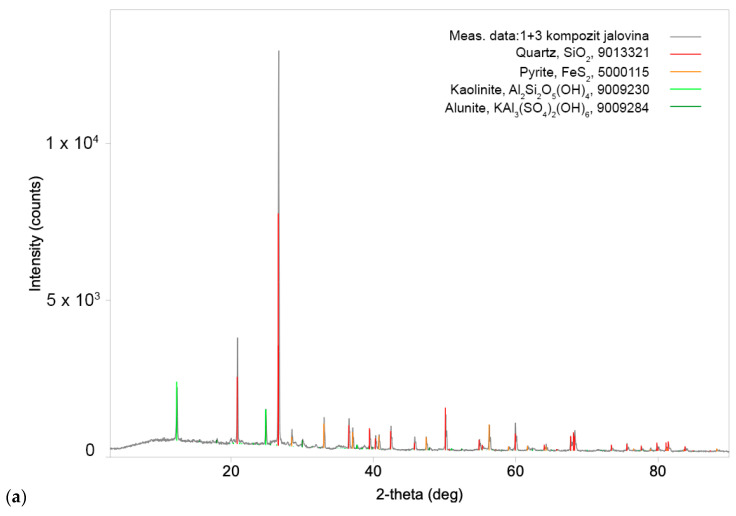

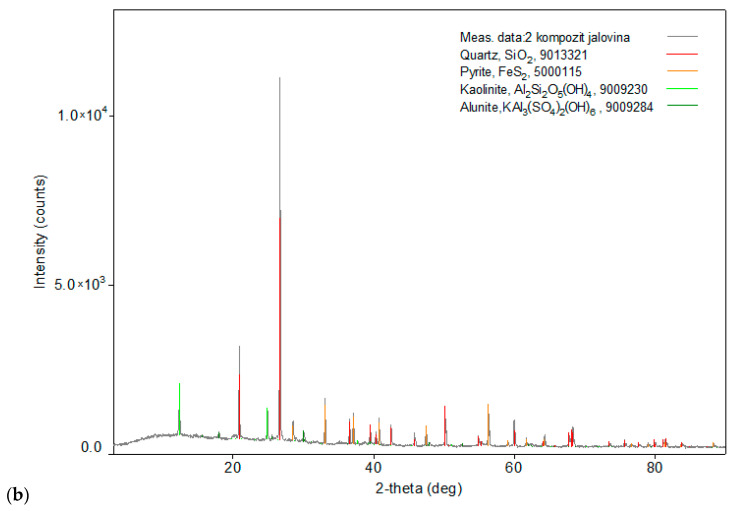
XRD spectra of representative as-received samples of (**a**) Composite I and (**b**) Composite II from the old flotation tailings dump of the Copper Mine Bor.

The results of the XRD analysis revealed nearly the same qualitative mineralogical content in both composites. Quartz (SiO_2_) is the most abundant, then pyrite (Fe_2_S) and kaolinite (Al_2_Si_2_O_5_(OH)_4_), while alunite (KAl_3_(SO_4_)_2_(OH)_6_) is the least abundant in both Composite I and Composite II. Diffractograms of Composite I and Composite II samples are shown in Figure 2a and Figure 2b, respectively.

### 3.4. Results of Cu Leaching—Experiment 1

The results of the leaching test, given in Table 6, suggest that the highest percentage of both copper leaching (89.34%) and iron leaching (65.15%) is achieved after 56 h of procedure. Also, a notably high level of copper leaching (88.80%) is gained after the first 8 h of leaching, unlike the significantly lower level of iron leaching (31,74%) after the same time compared to leachate amounts after 56 h of leaching. There was a slightly poorer mixing of gases noticed in the Composite II sample during the test due to a failure in the inlet valve, which caused a lower leaching rate of copper.

The pH values were constantly dropping over time during the experiment, indicating the occurrence of sulfur oxidation and sulfuric acid creation. On the other hand, the increase in redox potential during the leaching indicates the occurrence of iron leaching and Fe^3+^ ion formation.

The measured mass of the solid residue of the Composite I sample was 155.3 g, meaning that there was a 17.17% decrease in mass. The mass of the solid residue of the Composite II sample weighed 156.5 g, i.e., the mass decrease percentage was 16.53%.

### 3.5. Results of Cu Leaching—Experiment 2

From the results presented in Table 7, it is obvious that 88.80% of Cu and 17.45% of Fe were leached in only one hour from the Composite I sample. The copper leaching in 8 h is only slightly higher than that after 1 h, but the iron leaching is 2.5× more, which is regarded as undesirable. Therefore, an acceptable leaching time is 1 h. The slide valve for uniform gas distribution experienced a minor malfunction that led to a slightly lower Cu leaching rate in Composite II.

**Figure 3:**
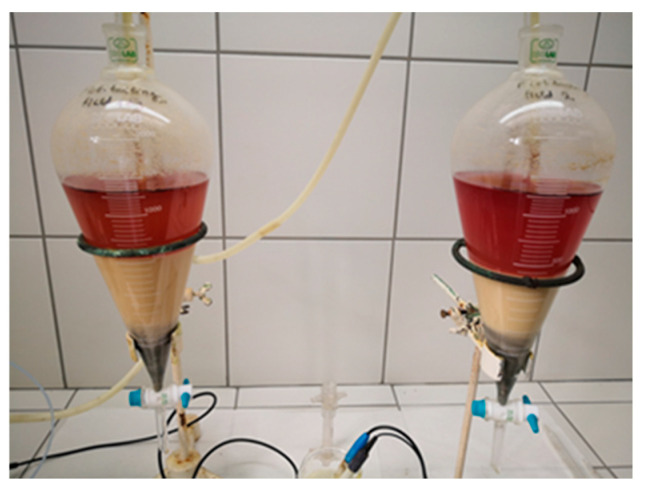
Separated phases after leaching of Composite I and Composite II samples.

After stopping the leaching process and phase separation, there appeared two phases: a liquid one (leaching solution) and a solid residue from leaching. Figure 3 represents the evidence of two solid phases after leaching: a heavier dark one and a lighter white one.

### 3.6. Results of XRD Characterization of Separated Phases

#### 3.6.1. XRD of Dark and White Precipitates from “Field I”

**Figure 4:**
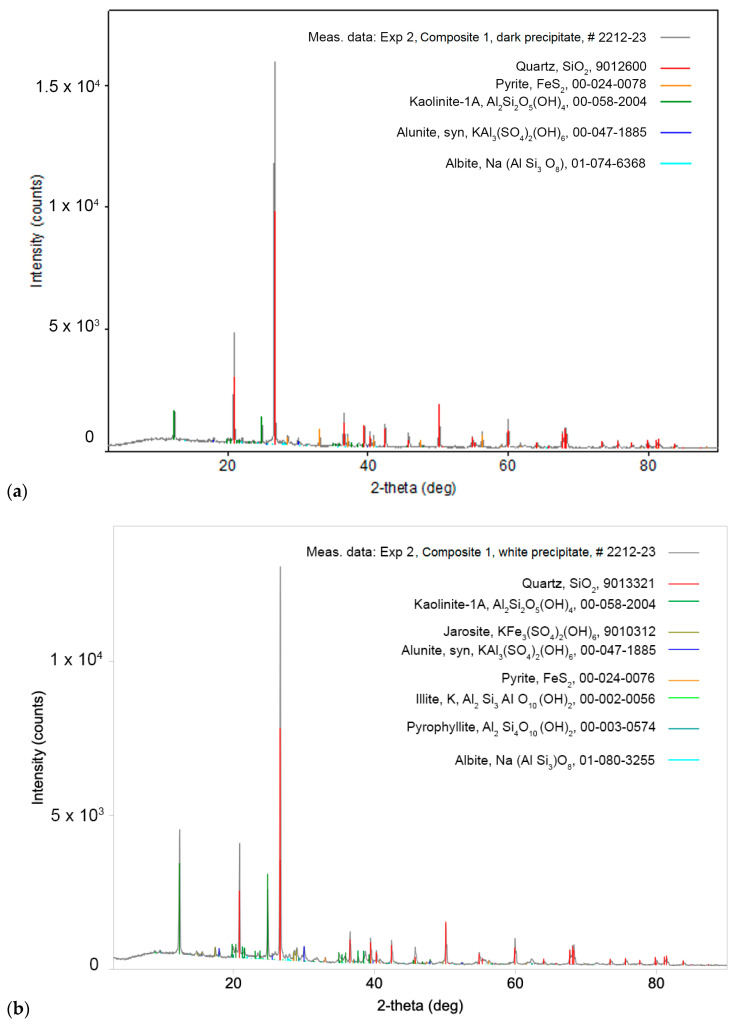
(**a**) XRD spectra of the dark precipitate of Composite I sample from experiment 2; (**b**) XRD spectra of the white precipitate of Composite I sample from experiment 2.

A diffractogram of the dark precipitate of the Composite I sample from experiment 2 is shown in Figure 4a. The following minerals have been identified in this sample: quartz (SiO_2_), kaolinite (Al_2_Si_2_O_5_(OH)_4_), pyrite (FeS_2_), alunite (KAl_3_(SO_4_)_2_(OH)_6_), and plagioclase (NaAlSi_3_O_8_–CaAl_2_Si_2_O_8_). The most abundant is quartz, followed by kaolinite and pyrite, while there is a low abundance of alunite and plagioclase.

The diffractogram of the white precipitate of Composite I sample from experiment 2 is shown in Figure 4b. The minerals identified in this sample were quartz (SiO_2_), kaolinite (Al_2_Si_2_O_5_(OH)_4_), jarosite (KFe_3_(SO_4_)_2_(OH)_6_), alunite (KAl_3_(SO_4_)_2_(OH)_6_), pyrite (FeS_2_), hydro-muscovite (KAlSi_3_AlO_10_(OH)_2_), phyllosilicate (Al_2_Si_4_O_10_(OH)_2_), and plagioclase (NaAlSi_3_O_8_–CaAl_2_Si_2_O_8_). The most abundant minerals are quartz and kaolinite, while jarosite, alunite, pyrite, hydro-muscovite, phyllosilicate, and plagioclase are less abundant.

#### 3.6.2. XRD of Dark and White Precipitates from “Field II”

**Figure 5:**
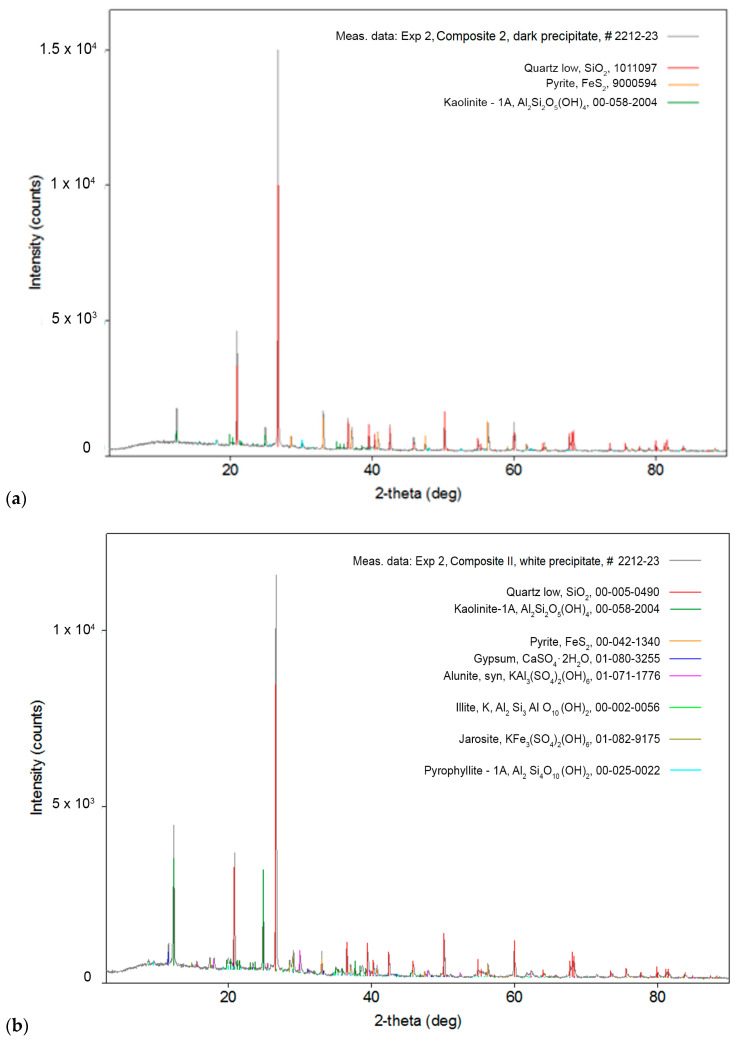
(**a**) XRD spectra of the dark precipitate of Composite II sample from experiment 2; (**b**) XRD spectra of the white precipitate of Composite II sample from experiment 2.

The XRD spectra of the dark precipitate of Composite II sample from experiment 2 are shown in Figure 5a. The following minerals were identified in this sample: quartz (SiO_2_), kaolinite (Al_2_Si_2_O_5_(OH)_4_), and pyrite (FeS_2_). The most abundant is quartz, less abundant are kaolinite and pyrite, while alunite is present in a very small amount.

The XRD spectra of the white precipitate of Composite II from experiment 2 are shown in Figure 5b. The minerals identified here were quartz (SiO_2_), kaolinite (Al_2_Si_2_O_5_(OH)_4_), pyrite (FeS_2_), gypsum (CaSO_4_·2H_2_O), alunite (KAl_3_(SO_4_)_2_(OH)_6_), hydro-muscovite (KAlSi_3_AlO_10_(OH)_2_), jarosite (KFe_3_(SO_4_)_2_(OH)_6_), and phyllosilicate (Al_2_Si_4_O_10_(OH)_2_). The most abundant are quartz and kaolinite, and pyrite, gypsum, alunite, and jarosite were less abundant, while there is very little hydro-muscovite and phyllosilicate in the sample.

### 3.7. SEM/EDS Characterization

#### 3.7.1. SEM/EDS of Precipitates from Composite I

**Figure 6:**
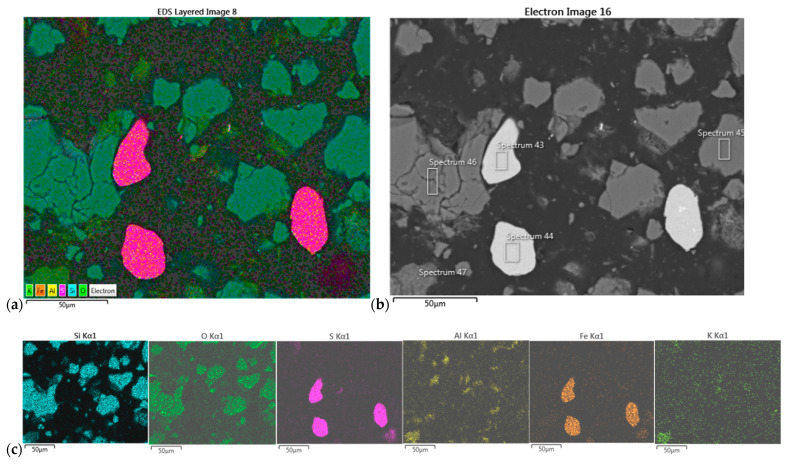
(**a**) EDS map-scan of the dark precipitate from Composite I sample and (**b**) SEM image of the sample (500× magnification) with labeled positions of EDS pointscan; (**c**) elemental distribution of mapscan.

The EDS map-scan of the dark precipitate from the Composite I sample revealed the intense presence of Fe and S, followed by Si and O and some Al and K. Based on the determined percentages in point-scan elemental analysis at the specific chosen spots, the following crystal structures have been assumed: FeS_2_ (pyrite), SiO_2_ (quartz), and mica (KFe_3_AlSi_3_O_10_(OH)_2_) as a minority (see Figure 6 and Table 8).

**Figure 7:**
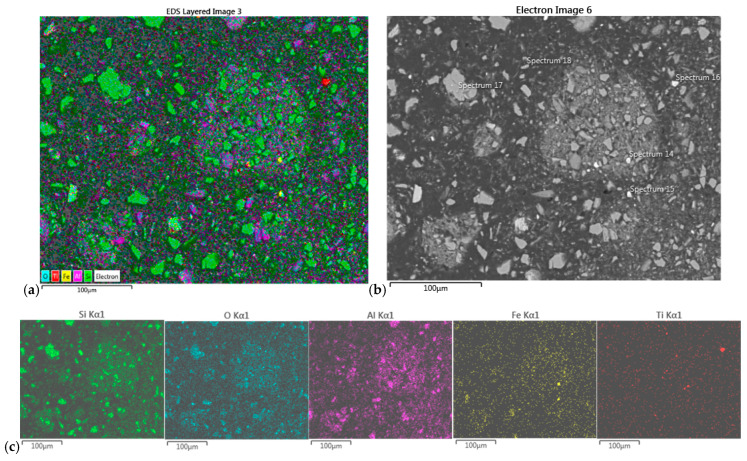
(**a**) EDS map-scan of the white precipitate from Composite I sample, and (**b**) SEM image of the sample (350× magnification) with labeled positions of EDS pointscan; (**c**) elemental distribution.

Similar to the dark precipitate, the EDS scan of the white precipitate from the Composite I sample again revealed the intense presence of Fe, S, Si, and O in four of the analyzed spots and some titanium (besides the mentioned elements) in the fifth spot. Based on obtained percentages in point-scan elemental analysis at the specific chosen spots, the crystal structures of FeS_2_, SiO_2_, and rutile (TiO_2_) have been determined in this precipitate (Figure 7 and Table 9).

The results showed rather uniform composition in both white and dark (i.e., light and dark) precipitates of the “Field I” sample, based mainly on pyrite and quartz.

#### 3.7.2. SEM/EDS of Precipitates from Composite II

**Figure 8:**
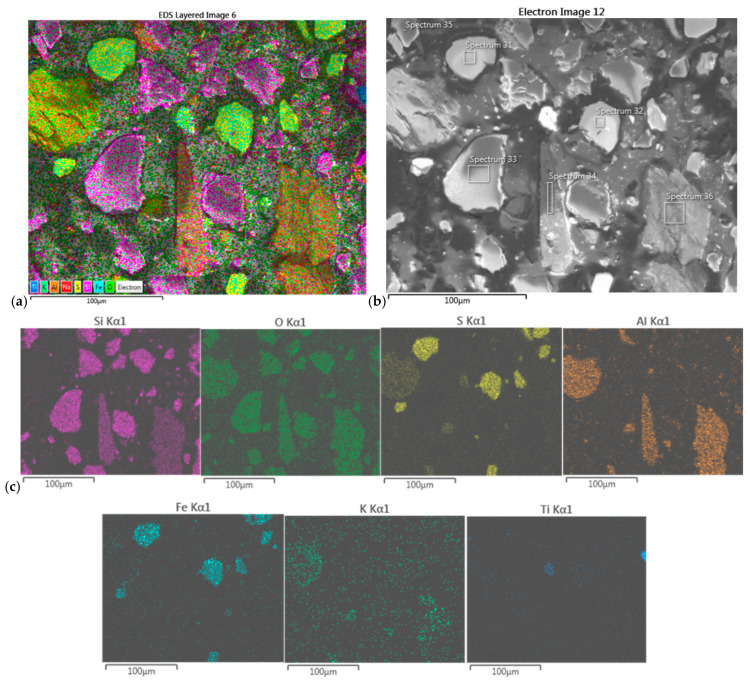
(**a**) EDS map-scan of the dark precipitate from Composite II sample, and (**b**) SEM image of the sample (500× magnification) with labeled positions of EDS pointscan; (**c**) elemental distribution.

The EDS map-scan of the dark precipitate from Composite II revealed the presence of Fe, S, Si, O, and Al, followed by some of Ti and K. Based on the percentages obtained in the point-scan elemental analysis at the specific chosen spots, the following crystal structures have been assumed: FeS_2_, SiO_2_, and Al_2_Si_2_O_5_(OH)_4_ (kaolinite) as a minority (see Figure 8 and Table 10).

**Figure 9:**
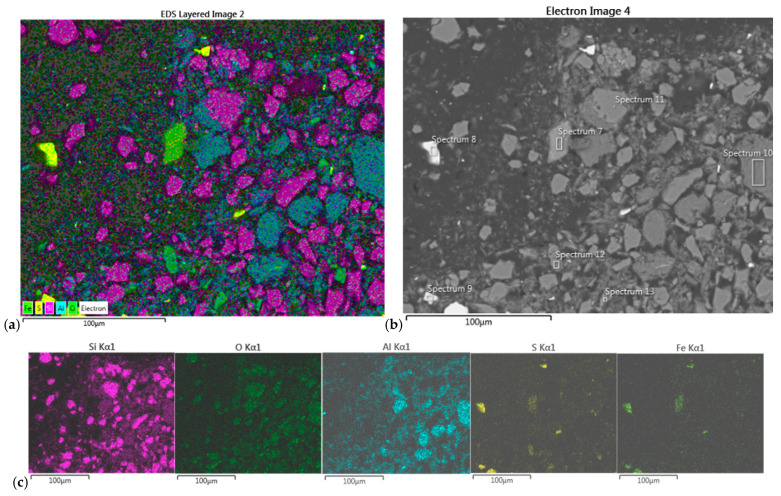
(**a**) EDS map-scan of the white precipitate from Composite II sample, and (**b**) SEM image of the sample (500× magnification) with labeled positions of EDS pointscan; (**c**) elemental distribution.

The EDS scan of the white precipitate from the Composite II sample confirmed the presence of Fe, S, Si, O, and Al and a little Na and K. The elemental percentages, obtained in the point-scan elemental analysis at the specific chosen spots, revealed the following crystal structures: FeS_2_, SiO_2_, Al_2_Si_2_O_5_(OH)_4_, and alunite (KAl_3_(SO_4_)_2_(OH)_6_), with some possible traces of KNaSiO_3_ (Figure 9 and Table 11).

The findings of the EDS scans of the two precipitates from both samples are consistent with the findings of XRD analyses, although less informative than XRD.

The chemical analysis of the Composite I sample revealed 0.3 g/t of Au from 46.4 g of the white precipitate and 0.8 g/t of Au from 46.4 g of the dark precipitate. In the Composite II sample, on the other hand, there was 0.3 g/t of Au out of 29.1 g of the white precipitate, while there was 0.5 g/t of Au in 60.8 g of the dark one.

### 3.8. Results of Au and Ag Leaching from Solid Residue

The outcome of leaching Au and Ag is given in Table 3, meaning that in the treatment of the solid residue after Cu leaching, the amounts of 83.42% Au and 11.55% Ag from Composite I and amounts of 79.12% Au and 13.63% Ag from Composite II have been leached (see Table 12).

## 4. Discussion

Ozone leaching leads to the destruction of pyrite crystal lattice and gold atoms released from the lattice. The evidence of the partial dissolution of pyrite also comes from the X-ray diffraction peak decrease after leaching. As gold has a high mass density, the greatest part of the gold has been concentrated in the dark precipitate. Since a significant amount of gold remained in the white phase, the separation of solid phases was mitigated, i.e., the entire solid phase was subjected to leaching.

Since few experiments were conducted, because of the small sample set size, neither the ozone consumption nor the utilization efficiency were accurately determined.

**Figure 10:**
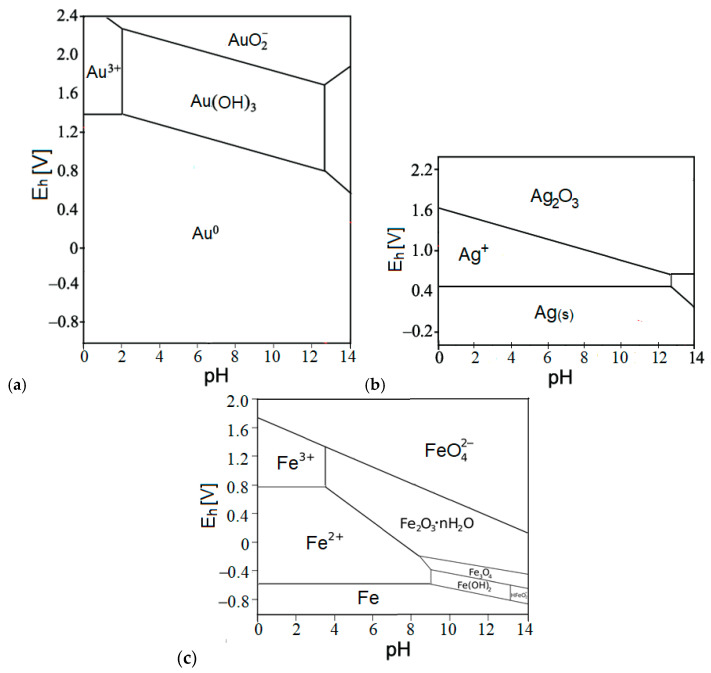
Pourbaix diagrams (E_h_ vs. pH) at 25 °C of: (**a**) Au-O system, (**b**) Ag-O system, and (**c**) Fe-O system (assumed dissolved concentration of Au, Ag and Fe, respectively: 10^−4^ molL^−1^) [46].

Based on the Pourbaix diagram calculated (Figure 10) at pH = 9 while the ozone was in surplus (and ozone oxidation potential being ~2.1 V), the forecasted most expected species would be AuO_2_¯ in the absence of chelating agents; however, the excess of thiosulfate anion captures Au^3+^ cation in solution, thus preventing the Au-oxide anion formation.

The recovery rate of silver is significantly lower than that of gold (11.55–13.63% vs. 83.42%). The dominating factors for this discrepancy are passivation and formation of insoluble compounds [27,35], represented in Pourbaix diagram (Figure 10b), where at or above the ozone oxidation potential of 2.1 V, the insoluble Ag_2_O_3_ species prevails, thus significantly disabling the thiosulfate complexation with Ag-ions, resulting in notably lower leaching rates for silver.

The activation energy of Ag-oxidation (53.1 kJ/mol) [47] indicates that the process of ozone diffusion is the main controlling step in the reaction, and the total reaction rate is governed by the diffusion rate, meaning the first-order kinetics. These results revealed that the O_3_ inlet rate (and therefore its concentration in the solution, too) is the pivotal factor in determining the leaching rate in the reaction system, while the thiosulfate addition preserves Ag^+^ and mediates to its elemental passivation prior to the Ag oxide or hydroxide formation.

**Figure 11:**
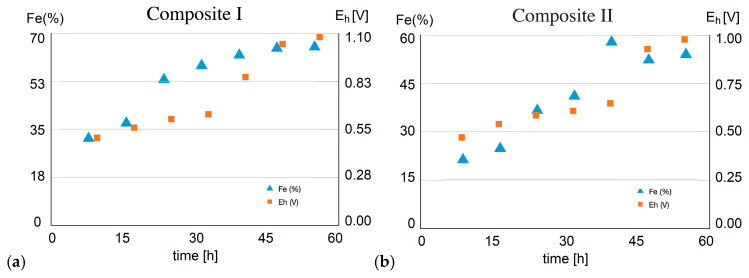
Correlations between iron gain and redox potential increase during the leaching of (**a**) Composite I, (**b**) Composite II.

There is a positive correlation between the change in redox potential and the kinetics of copper leaching (Figure 11), with Fe% uniformly increasing too (Table 6). The following four parameters are positively correlated and increase simultaneously over time: Cu%, Fe%, redox potential, and sulfuric acid amount. The redox potential increases as a consequence of pyrite oxidation by inletting ozone into ferric iron (Fe^3+^). On the other hand, copper sulfide minerals (bornite, covellite, chalcocite, and chalcopyrite) are oxidized by ferric iron. The oxidation of the pyrite is an acid generating reaction [9], which explains the drop in the pH value of the leach solution since pyrite is much more abundant in the flotation tailings in comparison to copper sulfide minerals [9]. During the oxidation of copper sulfide minerals, Fe^3+^ is being reduced back to ferrous iron (Fe^2+^), and the higher the concentration of Fe^3+^ ions over Fe^2+^ ions, the higher positive redox potential it creates (given by Pourbaix diagram for Fe-O system, Figure 10c). Also, intermediate sulfur species and competing reactions with ferrous ions might partially reduce the overall efficiency of copper dissolution. As a result, the reaction rate of chalcopyrite dissolution decreases with the increasing sulfuric acid concentration [48], reaching the saturation.

In Composite I, the copper leaching efficiency was notably high, starting at 88.80% after the first hour and reaching 89.00% by the eighth hour. This can be attributed to pyrite, which facilitates the oxidation process and promotes copper dissolution. Composite II exhibited lower copper leaching efficiency partly due to the inhibitory effects of gypsum (CaSO_4_ × 2H_2_O), which may act as a barrier to leaching.

However, the differences between Composite I and Composite II in copper and iron leaching efficiencies are mainly ascribed to the differences in total pyrite amount in composites before treatment and to two adverse (conflicting) effects of pyrite. The more pyrite is oxidized (into ferric), the more ferric iron is created, which promotes Cu oxidation and dissolution (leaching). However, the more pyrite is oxidized, the more sulfuric acid is subsequently created. And the more acidity (i.e., the less pH), the slower the copper dissolution process (i.e., reducing the overall leaching efficiency). The pH < 2 during the entire process of Cu and Fe leaching (Table 6), and the redox potential (E_h_) is above 0.8 V after some time (≥40 h for composite I, i.e., ≥48 h for composite II). Fe(II) in pyrite is oxidized by ozone into Fe(III) that exists dominantly in form of Fe^3+^ ion at pH < 2 and E_h_ > 0.8 V in aqueous solution (see the Pourbaix diagram of iron, Figure 10c). There was no detected precipitation of any iron compound in the leachate, mainly due to the low pH (<2). The E_h_ > 0.45 V (and increasing) during the entire leaching of both composites. Fe^2+^ is dominant below 0.8 V, and Fe^3+^ is dominant above it in the leachate.

Despite the differences in absolute copper leaching efficiency, the correlation coefficient between the two fields is still 0.92, meaning a good correlation. This indicates the dependence of leaching efficiency on the abundance of reactive minerals in composite, which facilitate copper dissolution, and this dependence demands further investigation. Also, the effects of certain minerals in composites (such as jarosite, alunite, etc.) on reagent consumption, metal recovery, and the overall leaching efficiency need a thorough examination in further studies.

For iron leaching, Composite I demonstrated higher efficiency, with percentages starting at 17.45% and reaching 44.81% by the eighth hour. This higher efficiency can be attributed to the presence of iron-bearing minerals such as pyrite and jarosite (KFe_3_(SO_4_)_2_(OH)_6_), which are known to release iron more readily under oxidative conditions. Composite II, however, showed slower iron leaching, with percentages starting at 11.12% and reaching 20.32% by the eighth hour. This reduced efficiency is linked to the lower abundance of iron-reactive minerals and the possible inhibitory effects of gypsum. Despite this, the correlation of 0.98 between iron leaching in the two fields indicates that the trends are very similar, suggesting that mineralogy rather than leaching kinetics drives the differences in efficiency.

The potential degradation of thiosulfate during the process could impact the efficiency of both Au and Ag leaching. At this point, the stability of thiosulfate has not been monitored and is going to be the subject of an upcoming study. Further investigations are planned to also focus on the following topics: (1) the kinetics of ozone oxidation of Au and Ag, (2) the relative contributions of Au⁺/Au^3^⁺ (and Ag⁺/Ag^3^⁺) to the overall leaching process, and (3) the stability of [Au(S_2_O_3_)_2_]^3−^ and [Ag(S_2_O_3_)_2_]^3−^ complexes (relative to pH and in presence of competing ions).

This method has outstanding economic and ecological benefits: the ozone leaching process of tailings lasts for a short time, will take place in simple tanks, does not produce SO_2_ gas and slugs, and works with diluted acid. Furthermore, it does not need heating and requires small financial investments for equipment and probably the low consumption of ozone, and therefore, the assumed energy consumption is low. For precise ozone consumption measurement, a larger laboratory scale and pilot plant are needed, where the contact between air-enriched ozone will be longer, therefore attaining better recovery rates.

## 5. Conclusions

Based on the leaching results of the Composite I and Composite II flotation tailings, the following conclusions could be made:

(1) By leaching the Composite I for 1 h under the given experimental conditions (solid-to-liquid phase ratio of 1:4, with water as leaching medium at room temperature with ozone gas inlet), the leaching of 88.80% Cu and 17.45% Fe can be attained.
(2) By leaching the Composite II under the same experimental conditions as Composite I, a lower leaching efficiency of 59.10% Cu and 11.12% Fe has been attained. Part of the cause of this was a minor fault in the slide valve for uniform gas distribution, which resulted in slightly lower Cu leaching.
(3) The solid residue, obtained after Cu leaching, was treated for leaching Au and Ag. The leaching of Au and Ag was 83.42% and 11.55%, respectively, at the optimum conditions (pH = 9, T = 25 °C, O_3_ inlet = 3 L/min at 80% capacity, Na_2_S_2_O_3_ addition = 50 g/L).
(4) The ozone consumption during the experiment was 12 g/h per sample. Further research is proposed to be conducted on larger sample size in order to more precisely determine the ozone consumption and utilization efficiency degree.

The proposed ozone-assisted leaching of Cu, Fe, Au, and Ag is a promising alternative for the eco-friendly recovery of valuable metals from mixed sulfide-oxide ores at room temperature and atmospheric pressure.

## Figures and Tables

**Table 1:** Basic physical and chemical properties of representative samples from two flotation tailings’ composites.

Samples	Apparent Density of Particles (g/cm^3^)	Bulk Density (kg/m^3^)	pH
Composite I	2.765	1176.0	2.82
Composite II	2.690	976.0	2.77

**Table 2:** Granulometric composition of the sample of Composite I flotation tailings.

Sieve Hole Diameter d (mm)	Mass Participation m (%)	Cumulative Retaining R (%)	Cumulative Passing D (%)
−4.00 + 2.36	7.6	7.6	100.0
−2.36 + 1.70	0.2	7.8	92.4
−1.70 + 0.85	0.4	8.2	92.2
−0.85 + 0.60	0.4	8.6	91.8
−0.60 + 0.42	0.3	8.9	91.4
−0.42 + 0.30	6.7	15.6	91.1
−0.30 + 0.21	1.1	16.7	84.4
−0.21 + 0.15	4.4	21.1	83.3
−0.150 + 0.106	6.3	27.4	78.9
−0.106 + 0.075	6.1	33.5	72.6
−0.075 + 0.053	5.5	39.0	66.5
−0.053 + 0.038	5.5	44.5	61.0
−0.038 + 0.000	55.5	100.0	55.5

**Table 3:** Granulometric composition of the sample of Composite II flotation tailings.

Sieve Hole Diameter d (mm)	Mass Participation m (%)	Cumulative Retaining R (%)	Cumulative Passing D (%)
−4.00 + 2.36	0.3	0.3	100.0
−2.36 + 1.70	0.1	0.4	99.7
−1.70 + 0.85	0.1	0.5	99.6
−0.85 + 0.60	0.1	0.6	99.5
−0.60 + 0.42	0.1	0.7	99.4
−0.42 + 0.30	0.4	1.1	99.3
−0.30 + 0.21	7.2	8.3	98.9
−0.21 + 0.15	6.3	14.6	91.7
−0.150 + 0.106	8.6	23.2	85.4
−0.106 + 0.075	7.7	30.9	76.8
−0.075 + 0.053	7.8	38.7	69.1
−0.053 + 0.038	6.2	44.9	61.3
−0.038 + 0.000	55.1	100.0	55.1

**Table 4:** Chemical content of representative composite samples from two flotation tailings.

Element	Content (%)		Analytical Method
	Composite I	Composite II	
Cu_tot_	0.25	0.23	AAS
Cu_ox_	0.18	0.12	
Au	0.45	0.40	
Ag	0.00014	0.0002	
Fe	7.62	8.90	ICP-AES
Ca	0.78	0.74	
K	0.54	0.59	
Na	0.23	0.19	
Al_2_O_3_	10.8	11.0	
Sr	0.065	0.064	
As	0.012	0.017	
Zn	0.0024	0.0049	
S	9.34	12.4	ACS
SiO_2_	52.9	49.9	TGA

**Table 5:** Qualitative mineralogical analysis of the composite samples.

Mineral	Composite I (%)	Composite II (%)
Pyrite	17.15	22.96
Covelline	0.02	0.12
Digenite	0.0	0.10
Enargite	0.34	0.08
Tetrahedrite	0.05	0.05
Chalcopyrite	0.09	0.02
Chalcocite	0.04	< 0.01
Native gold	<0.01	-
Magnetite	0.32	0.19
Hematite	0.11	0.04
Rutile	0.21	0.27
Leucoxene	0.31	0.61
Cassiterite	0.0	0.03
Cu-limonite	0.90	0.72
Tailings minerals	80.46	74.81
Total:	100.00	100.00

**Table 6:** Leaching percentage of Cu and Fe, pH value, and redox potential (E_h_) during the leaching process on Composite I and Composite II samples.

Time	Composite I				Composite II			
	Cu(%)	Fe(%)	pH	E_h_ (mV)	Cu(%)	Fe(%)	pH	E_h_ (mV)
8 h	88.80	31.74	1.80	502	60.50	21.29	1.7	470
16 h	88.85	37.35	1.50	560	62.20	24.79	1.6	540
24 h	88.92	53.18	1.45	609	65.61	36.72	1.50	585
32 h	89.00	58.30	1.35	637	70.72	41.12	1.34	608
40 h	89.12	62.19	1.30	850	72.86	58.01	1.29	648
48 h	89.13	64.71	1.20	1040	75.70	52.41	1.19	930
56 h	89.34	65.15	1.19	1080	77.44	54.16	1.17	980

**Table 7:** Leaching percentage of Cu and Fe during the leaching of Composite I and Composite II samples from flotation tailings.

Time (h)	Composite I		Composite II	
	Cu(%)	Fe(%)	Cu(%)	Fe(%)
1	88.80	17.45	59.10	11.12
2	88.82	18.20	60.87	13.26
4	88.85	28.95	63.30	16.06
6	88.90	37.55	64.52	18.89
8	89.00	44.81	66.17	20.32

**Table 8:** Chemical composition and identified crystal structures of dark precipitate from Composite I, based on EDS spectra.

Spectrum #	Elements (%)						Crystal
	O	Al	Si	S	K	Fe	
Spectrum 43				54.74		45.26	FeS_2_
Spectrum 44				55.02		44.98	FeS_2_
Spectrum 45	52.51		47.49				SiO_2_
Spectrum 46	53.31		46.69				SiO_2_
Spectrum 47	48.28	17.62	26.34		6.57	1.19	mica

**Table 9:** Chemical composition and identified crystal structures of white precipitate from Composite I, based on EDS spectra.

Spectrum #	Elements (%)					Compound
	O	Si	S	Ti	Fe	
Spectrum 14			54.37		45.63	FeS_2_
Spectrum 15			55.07		44.93	FeS_2_
Spectrum 16	40.39			59.61		TiO_2_
Spectrum 17	52.06	47.94				SiO_2_
Spectrum 18	51.98	48.02				SiO_2_

**Table 10:** Chemical composition and identified crystal structures of dark precipitate from Composite II, based on EDS spectra.

Spectrum #	Elements (%)							Crystal
	O	Al	Si	S	K	Ti	Fe	
Spectrum 31				59.18		1.1	39.72	FeS_2_ with Ti
Spectrum 32				62.17			37.83	FeS_2_
Spectrum 33	60.45		39.55					SiO_2_
Spectrum 34	58.67	14.15	27.18					Al_2_Si_2_O_5_(OH)_4_
Spectrum 35	65.43		34.57					SiO_2_
Spectrum 36	60.93	18.7	19.34		1.03			Al_2_Si_2_O_5_(OH)_4_

**Table 11:** Chemical composition and identified crystal structures of white precipitate from Composite II, based on EDS spectra.

Spectrum #	Elements (%)							Compound
	O	Na	Al	Si	S	K	Fe	
Spectrum 7	42.63	1.9	0.48	1.38	15.96	2.61	35.03	FeS_2_ + KNaSiO_3_
Spectrum 8					54.86		45.14	FeS_2_
Spectrum 9					53.78		46.22	FeS_2_
Spectrum 10	53.75		22.41	23.84				Al_2_Si_2_O_5_(OH)_4_
Spectrum 11	51.71			48.29				SiO_2_
Spectrum 12	54.73	2.52	21.04		16.3	5.4		KAl_3_(SO_4_)_2_(OH)_6_
Spectrum 13	57.35	2.26	20.24		14.9	5.26		KAl_3_(SO_4_)_2_(OH)_6_

**Table 12:** Leaching percentage of Au and Ag from solid residue.

Time	Field I		Field II	
	Au(%)	Ag(%)	Au(%)	Ag(%)
48 h	83.42	11.55	79.12	13.63

## Data Availability

Data are contained within the article.
